# NAVIGATING POST-ACUTE CARE: LIVED EXPERIENCES OF MINORITIZED OLDER ADULTS

**DOI:** 10.1093/geroni/igae098.4028

**Published:** 2024-12-31

**Authors:** Dominique Como

**Affiliations:** University of Pittsburgh, Pittsburgh, Pennsylvania, United States

## Abstract

Annually, over 40% of Medicare beneficiaries use post-acute care (PAC) after hospitalization to aid functional and medical recovery. Disparities in PAC access, care quality, and patient outcomes are evident across social identities (e.g., gender, ethnicity) and social determinants of health indicators (e.g., housing, food access). However, limited research has delved into the lived experiences of older adults using PAC services. Understanding barriers and facilitators from the patient perspective is crucial for addressing health disparities. This study explored the lived experiences of older adults in PAC settings. As part of a mixed-methods study, older adults (aged 55 or older) who self-identified as Black/African American or Hispanic/Latino/Spanish origin and were actively receiving care in a PAC facility in Southern California were invited to participate in interviews. A semi-structured interview guide was developed based on information obtained from a literature review and revised following content review from community member expert. Eight participants were interviewed between May and July 2024. Thematic analysis revealed four key themes, highlighting salient areas, influencing post-acute experiences: Patient Involvement in Healthcare Experiences (e.g., active participation, patient autonomy, and self-advocacy in healthcare decisions); Provider Competence & Attitude (e.g., provider communication, knowledge, and disposition); Care Delivery Considerations (e.g., staffing levels, time constraints, physical handling practices); and Support Networks & Resources (e.g., familial support, facility resources). These findings offer valuable insights for enhancing PAC patient health and satisfaction, by providing comprehensive high-quality care, improving the effectiveness of care delivery, and strengthening patient-provider interactions to better serve minoritized older adults.

